# The Role of Hydrogen Sulfide on Cardiovascular Homeostasis: An Overview with Update on Immunomodulation

**DOI:** 10.3389/fphar.2017.00686

**Published:** 2017-09-26

**Authors:** Li-Long Pan, Ming Qin, Xin-Hua Liu, Yi-Zhun Zhu

**Affiliations:** ^1^Department of Pharmacology, School of Pharmacy, Fudan University, Shanghai, China; ^2^State Key Laboratory of Quality Research in Chinese Medicine and School of Pharmacy, Macau University of Science and Technology, Macau, China

**Keywords:** myocardial ischemia, heart failure, atherosclerosis, inflammation, hydrogen sulfide

## Abstract

Hydrogen sulfide (H_2_S), the third endogenous gaseous signaling molecule alongside nitric oxide (NO) and carbon monoxide, is synthesized by multiple enzymes in cardiovascular system. Similar to other gaseous mediators, H_2_S has demonstrated a variety of biological activities, including anti-oxidative, anti-apoptotic, pro-angiogenic, vasodilating capacities and endothelial NO synthase modulating activity, and regulates a wide range of pathophysiological processes in cardiovascular disorders. However, the underlying mechanisms by which H_2_S mediates cardiovascular homeostasis are not fully understood. This review focuses on the recent progress on functional and mechanistic aspects of H_2_S in the inflammatory and immunoregulatory processes of cardiovascular disorders, importantly myocardial ischemia, heart failure, and atherosclerosis. Moreover, we highlight the challenges for developing H_2_S-based therapy to modulate the pathological processes in cardiovascular diseases. A better understanding of the immunomodulatory and biochemical functions of H_2_S might provide new therapeutic strategies for these cardiovascular diseases.

## Introduction

Cardiovascular diseases, the leading cause of death worldwide, are multifactorial resulting from disorders of the heart and circulation (Murphy et al., 2015), which cause immense health and economic burdens in all countries (Benjamin et al., 2017). The main risk factors associated with cardiovascular diseases are unhealthy lifestyle and lack of physical activity (American Heart Association Nutrition Committe et al., 2006). Accumulating evidence has demonstrated that the excess risk of cardiovascular outcomes is associated with changing endogenous H_2_S levels (Yang et al., 2008; Kondo et al., 2013; Mani et al., 2013; Wallace and Wang, 2015).

H_2_S, a colorless gas with characteristic rotten egg smell, is recognized an environmental hazard and a toxic agent for long. In addition, before the discovery that H_2_S is present in most organ systems in mammals including humans, it was believed that the gas was a byproduct of metabolic processes by microbes in the atmosphere. The pioneering study by the neuroscientist Hideo Kimura demonstrated that H_2_S at the physiological concentration facilitated hippocampal long-term potentiation in the nervous system, proposing that this gasotransmitter acts as a neuromodulator (Abe and Kimura, 1996). Later, H_2_S has become recognized widely as the third endogenous gaseous mediator alongside NO and CO for its modulatory effects on many signaling molecules, including kinases, phosphatases, and transcription factors (Abe and Kimura, 1996; Wang, 2002; Sun et al., 2011). Subsequently, it has been found to regulate both physiological and pathophysiological processes but at specific concentrations. Over the past decade, H_2_S has been found to be synthesized primarily through metabolic processes from cysteine and homocysteine in a variety of tissues where it functions as a signaling molecule (Szabo, 2007). H_2_S exerts its cellular effects by directly transport across cell membranes by simple diffusion without the need of specific membrane receptors and it is also involved in the modulation of many pathophysiological processes in cardiovascular system (Chunyu et al., 2003; Mathai et al., 2009; Wang et al., 2009; Liu et al., 2012; Pan et al., 2012). So far, a plethora of investigations have been performed on therapeutic values of H_2_S in cardiovascular diseases, which reveal that H_2_S at physiological levels has an important role in cardiovascular homeostasis, and inhibitors of endogenous H_2_S production or H_2_S donors exert significant effects in cardiovascular diseases, including heart failure, ischemic myocardium, atherosclerosis, and hypertension (Yang et al., 2008; Wang et al., 2009; Calvert et al., 2010; Miao et al., 2016a). Furthermore, empirical studies have elucidated several mechanisms of H_2_S-mediated cardiovascular protective activities, which include, but are not restricted to, anti-oxidation (Chang et al., 2008; Wang et al., 2009; Huang et al., 2017), anti-apoptosis (Yan et al., 2017), ion channel regulation (Pan et al., 2008; Ma et al., 2015), pro-angiogenesis (Cai et al., 2007), and anti-inflammation (Pan et al., 2011; Wallace and Wang, 2015). The realization of biological importance of H_2_S in numerous cells, tissues and organs is now shedding light on the pathogenesis of various cardiovascular diseases, and paving the way for innovative therapeutic interventions (Wallace and Wang, 2015; Zheng et al., 2017). Meanwhile, regulation of H_2_S functions during cardiovascular diseases remains to be better understood.

## The Modulation of Endogenous H_2_S Biosynthesis in Cardiovascular System

**FIGURE 1:**
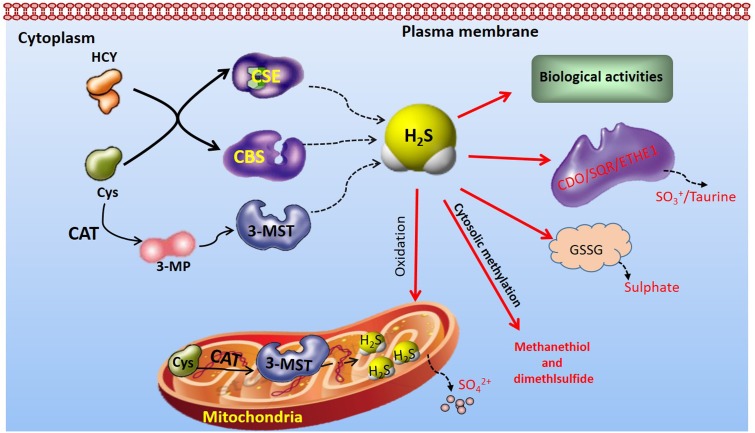
Overview of H_2_S-producting enzymes distribution and endogenous H_2_S metabolism CBS and CSE, distributed in the cytoplasm, produce H_2_S from Cys alone or from cysteine with HCY. In contrast, endogenous H_2_S production by 3-MST is primarily from 3-MP, which is produced from cysteine and α-ketoglutarate by CAT. 3-MST is distributed in both mitochondria and cytoplasm. The endogenous H_2_S is inactivated in the biological systems by the enzymes ETHE1, SQR, and CDO, or by means of mitochondrial oxidation, cytosolic methylation, scavenging by GSSG or other metallo- or disulfide-containing molecules. 3-MP, 3-mercaptopyruvate; 3-MST, 3-mercaptopyruvate sulfurtransferase; CAT, cysteine aminotransferase; CBS, Cystathionine β-synthase; CDO, cysteine dioxygenase; CSE, cystathionine γ-lyase; Cys, cysteine; HCY, homocysteine; ETHE1, ethylmalonic encephalopathy protein 1; GSSG, glutathione disulfide; SQR, sulfur:quinone oxidoreductase.

Endogenous H_2_S is produced in mammalian tissues by primarily enzymatic or non-enzymatic pathways (Li et al., 2011; Martelli et al., 2012; Pan et al., 2012; Mani et al., 2014). Current understanding of H_2_S biology has arisen mostly from research work focused on enzymatic pathways (Liu et al., 2012; Wallace and Wang, 2015). The majority of endogenous H_2_S is synthesized by CBS (Szabo, 2007), CSE (Zhao et al., 2001), and 3-MST (Shibuya et al., 2009a,b). CBS and CSE may produce H_2_S from cysteine alone or from cysteine with homocysteine (Kimura, 2015). 3-MST produces endogenous H_2_S from one of the following substrates: 3-MP by CAT, thioredoxin, dihydrolipoic acid and D-cysteine along with DAO (Mikami et al., 2011; Yadav et al., 2013). All three H_2_S-synthesizing enzymes have been reported to be expressed by cardiovascular cells (Yang and Wang, 2015) (**Figure 1**). The distribution of H_2_S-producing enzymes in mammalian tissues is tissue-specific (Pan et al., 2012). CBS expression predominates in the brain, nervous system, liver, and kidneys, while CSE is a major H_2_S-synthesizing enzyme present in cardiovascular system under normal physiological conditions (Yang and Wang, 2015). 3-MST, along with CAT, accounts for H_2_S production in vascular endothelium in cardiovascular system (Shibuya et al., 2009a). In addition to their tissue-specific distribution, the intracellular localization of the three enzymes is different (Pan et al., 2012; Donnarumma et al., 2017). While CBS and CSE are cytosolic enzymes (Pan et al., 2012), 3-MST is present in both the mitochondrial and the cytosol with approximately two thirds of 3-MST found in the mitochondria (Li et al., 2009). Despite these findings, there is no definitive information regarding the relative contributions of each of the three enzymes on circulating and tissue H_2_S levels (Donnarumma et al., 2017). To maintain an appropriate physiological balance of H_2_S metabolism, endogenous H_2_S is inactivated in the biological systems by the enzymes ETHE1, SQR, and CDO, or by means of mitochondrial oxidation, cytosolic methylation, scavenging by glutathione disulfide or other metallo- or disulfide-containing molecules, as well as by release from the lungs (Mani et al., 2014; Donnarumma et al., 2017; Rose et al., 2017).

The critical roles of H_2_S at its physiologically relevant concentrations on the cardiovascular homeostasis have been well documented (Sun et al., 2011; Liu et al., 2012; Pan et al., 2012; Wang R. et al., 2015; Nagpure and Bian, 2016). The H_2_S levels in plasma and in organ tissues are regulated strictly by its generation and consumption under physiologic conditions (Liu et al., 2012). The H_2_S concentrations vary in different cells, tissues and organs and maintained within a certain range (Pan et al., 2012). Physiological levels of H_2_S range from 15 nM to 300 μM *in vivo* and the wide range of H_2_S levels may be due to variable detection methods used and the tissues analyzed (Liu et al., 2012; Hackfort and Mishra, 2016). Notably, significant changes of endogenous H_2_S levels (change of H_2_S-producing enzyme expression or its activity) have been clearly correlated to the pathogenesis of cardiovascular diseases, including heart failure, myocardial ischemia and atherosclerosis as indicated by both experimental and clinical evidence (Jiang et al., 2005; Feng et al., 2015; Gao et al., 2015; Wang W. et al., 2015; Yang and Wang, 2015; Kanagy et al., 2017; Li et al., 2017). Supplementation with exogenous H_2_S or modulation of endogenous H_2_S production markedly attenuates myocardial injury and improves cardiac function. For example, administration of SG1002 (a novel H_2_S prodrug, 400 mg) is reported to result in increased H_2_S levels and circulating NO bioavailability, and decreased circulating natriuretic peptide levels in patients with heart failure (Polhemus et al., 2015).

## Immuneregulatory Effects of H_2_S in Cardiovascular Diseases

The physiological and biomedical importance of H_2_S has been recognized in the cardiovascular homeostasis. Accumulating evidence has demonstrated the beneficial effects of H_2_S-based therapies in cardiovascular disorders, including atherosclerosis, ischemic and heart diseases, which have been well addressed earlier by high-quality reviews (Szabo, 2007; Jin et al., 2010; Liu et al., 2012; Wang, 2012; Polhemus and Lefer, 2014). The roles of H_2_S in modulating inflammatory and immune processes during cardiovascular diseases have been emerging. H_2_S has been shown to regulate various immune cell functions, such as T-cell activation and proliferation, monocyte and polymorphonuclear cell apoptosis, leukocyte adhesion and infiltration, and inflammatory cytokine release by immune cells. Recent evidence has highlighted that H_2_S also actively regulates immuno-inflammatory processes in cardiovascular diseases. The current review focuses on immune-inflammatory modulation in H_2_S-mediated cardiovascular homeostasis in conditions including myocardial ischemia, heart failure, and atherosclerosis.

## Myocardial Ischemia

Myocardial infarction is the leading cause of death worldwide with a yearly incidence of 1 million cases (Mozaffarian et al., 2015). During the deprivation of oxygen-carrying blood, combined with nutrient starvation, cardiomyocytes become insensitive to oxygen, leading to MI (Ghaderi et al., 2017). For those patients who undergo acute MI, the most effective therapeutic intervention is timely and effective myocardial reperfusion *via* revascularization combined with routine medical therapy (Hausenloy and Yellon, 2013). However, myocardial reperfusion can itself trigger cardiomyocyte death, an important complication of reperfusion therapy for MI, known as myocardial reperfusion injury (Boag et al., 2017). To date, no effective treatment has been identified. Among the pathological mechanisms underlying MI/R injury, inflammation and inflammatory cell infiltration, together with the activation of innate and adaptive immune responses, are the hallmarks of MI and reperfusion injury (Yellon and Hausenloy, 2007; Nahrendorf et al., 2010; Coggins and Rosenzweig, 2012; Frangogiannis, 2012; Swirski and Nahrendorf, 2013; Liu et al., 2016). Accumulating evidence suggests that modulation of excessive inflammation activation by negative regulation of toll-like receptor signaling and recruitment of inflammatory cells represents a promising therapeutic approach for MI and reperfusion injury (Yellon and Hausenloy, 2007; Coggins and Rosenzweig, 2012).

As the third endogenous gasotransmitter, H_2_S has emerged as an important mediator in maintaining cardiovascular homeostasis. Our group demonstrated for the first time that decreased plasma H_2_S levels were associated with increased infarct size and mortality. Administration of sodium hydrogen sulfide (NaHS, an exogenous H_2_S donor) decreased the infarct size of the left ventricle and MI-associated mortality in rats (Zhu et al., 2007). However, the changes of H_2_S levels during myocardial ischemia are still controversial. Ali et al. (2016) observed that serum H_2_S was significantly increased in ST-elevation acute MI patients. Li et al. (2016) have shown that plasma H_2_S levels decreased after acute MI surgery in rats. This observation is consistent with the previous study that either exogenous H_2_S administration or modulation of endogenous H_2_S production reduced myocardial ischemia-reperfusion injury in experimental models through restoration of H_2_S levels after ischemia (Elrod et al., 2007). The controversial results on serum H_2_S levels in ischemic myocardium cannot be readily explained by the different detection methods or the different species tested. However, the inconsistent results of serum H_2_S indeed merit further investigations.

**FIGURE 2:**
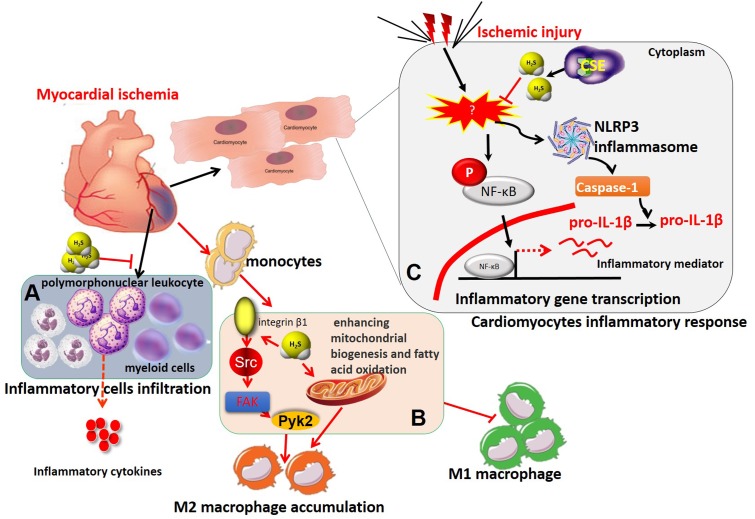
The immunomodulatory role of H_2_S in myocardial ischemia. **(A)** H_2_S treatment protected against ischemic myocardial injury by suppressing the recruitment of CD11b^+^ Gr-1^+^ myeloid cells and polymorphonuclear leukocytes to the ischemic myocardium and subsequent release of inflammatory cytokines, including TNF-α, IL-1β, and so on. **(B)** H_2_S recruited macrophages and induces M2 macrophage polarization in myocardial infarction by integrin β1-Src-FAK/Pyk2-Rac pathway and enhancing mitochondrial biogenesis and fatty acid oxidation. **(C)** H_2_S inhibited activation of NF-κB and NLRP3 inflammasome, subsequent inflammatory mediator expression and inflammatory responses in ischemia-stimulated cardiomyocytes. IL-1β, interleukin-1β; NLRP3, nucleotide-binding domain, leucine-rich-containing family, pyrin domain-containing-3; TNF-α, tumor necrosis factor-α.

In myocardium, enhanced H_2_S levels, whether by H_2_S supplementation or increased endogenous H_2_S production, have been found to protect the heart against ischemic injury. The exact cardioprotective mechanism of H_2_S has yet to be clarified but a number of molecular mechanisms have been identified, including vasodilation, anti-inflammation, antioxidation, anti-apoptosis, and modulation of cellular metabolism (Pan et al., 2012). MI and reperfusion injury trigger a complex immune-inflammatory responses in the injured myocardium, including inflammatory leukocyte infiltration and release of cytokines, such as IL-6, IL-8 and TNF-α. Recently, several studies, including our own, have demonstrated that H_2_S plays an important role in immune-inflammatory processes during MI and reperfusion injury (Elrod et al., 2007; Sodha et al., 2009; Miao et al., 2016a,b) (**Figure 2**). Neutrophils and leukocytes migrate into the infarcted myocardium during the first few hours after the onset of ischemia and peak after 1 day (Yellon and Hausenloy, 2007). Exogenous H_2_S administration or overexpression of the H_2_S-producing enzyme CSE significantly decreased leukocytes and neutrophil infiltration within the ischemic zone and markedly reduced myocardial inflammatory cytokine production during MI and reperfusion injury (Elrod et al., 2007). Additionally, exogenous H_2_S therapy was shown to attenuate cardiomyocyte apoptosis in the AAR of heart in a rat model of MI/R, whereas it decreased polymorphonuclear leukocyte accumulation and inflammatory mediators in the AAR from rat hearts subjected to regional MI/R (Sivarajah et al., 2009). Meanwhile, recruited monocytes/macrophages persist for days in the infarct area and contribute to inflammation, phagocytosis, proteolysis, angiogenesis, and collagen deposition (Nahrendorf et al., 2010). Modulated macrophage infiltration decreased inflammation, diminished interstitial fibrosis and improved cardiac remodeling and dysfunction (Nahrendorf et al., 2010). In a murine MI model subjected to pre- and post-coronary artery occlusion, exogenous H_2_S treatment reduced the recruitment of CD11b^+^ Gr-1^+^ myeloid cells to the myocardium, inhibited their migration from the splenic reservoir, and decreased serum TNF-α and IL-1β levels, thereby protecting against ischemic myocardial injury (Zhang et al., 2014). Recently, our study demonstrated that exogenous H_2_S treatment increased macrophage infiltration into the infarcted myocardium at the early stage of MI in both wild type and CSE-deficient mice (Miao et al., 2016b). In this study, exogenous H_2_S treatment promoted the migration of macrophages *in vitro*. Meanwhile, exogenous H_2_S treatment induced the activation of phosphor-Src, -Pyk2, -FAK^397^, and -FAK^925^. Moreover, exogenous H_2_S treatment induced internalization of integrin β1 on macrophage surface and promoted migration of macrophages and activation of Src signaling (Miao et al., 2016b). In our very recent study, we further demonstrated that exogenous H_2_S treatment ameliorated post-MI pathological cardiac remodeling and dysfunction in wild-type and CSE-deficient mice, decreased infarct size and mortality, and promoted M2 polarization of macrophages at the early stage of MI (Miao et al., 2016a). Notably, adoptive transfer of exogenous H_2_S-treated bone marrow-derived macrophages into wild-type and CSE-deficient mice with depleted macrophages also improved MI-induced cardiac dysfunction. A similar profile was also observed by Ji et al. (2017) that exogenous H_2_S treatment promoted microglia switch from a pro-inflammatory M1 phenotype to the modulatory M2 phenotype in ischemic stroke mice. Further mechanistic investigations demonstrated that exogenous H_2_S-induced M2 polarization of macrophages was achieved by enhanced mitochondrial biogenesis and fatty acid oxidation (Miao et al., 2016a).

In addition to regulatory effects of H_2_S on immune cell infiltration and phenotype switch, it also directly inhibits inflammatory responses in ischemic myocardium. For example, we found that *S*-propargyl-cysteine (SPRC, a novel endogenous H_2_S modulator) markedly attenuated LPS-induced TNF-α, ICAM-1, and iNOS expression in cardiomyocytes through modulation of CSE/H_2_S pathway by impairing inhibitory κBα (IκBα)/nuclear factor-κB (NF-κB inflammatory signaling and by activating PI3K/Akt signaling pathway (Pan et al., 2011). In addition, Toldo et al. (2014) revealed that Na_2_S (a H_2_S donor) administration during MI/R *in vivo* or *in vitro* prevented the activation of nucleotide-binding domain, leucine-rich-containing family, pyrin domain-containing-3 (NLRP3) inflammasome and caspase-1, a macromolecular complex responsible for sensing tissue injury or ‘danger’ and amplifying the inflammatory responses. Moreover, the NLRP3 inflammasome-inhibiting effects of H_2_S were completely abolished with deletion of microRNA-21, demonstrating that H_2_S suppressed myocardial inflammatory responses by inhibition of NLRP3 inflammasome activation and was dependent on microRNA-21 (Toldo et al., 2014) (**Figure 2**).

## Heart Failure

Heart failure is an inability of the heart to adequately meet the metabolic needs of the body, a clinical disease causing significant morbidity and mortality. Heart failure is the final outcome of conditions with varying etiologies. Atherosclerosis, risk factors, and comorbidities such as diabetes and obesity, many of which have an inflammatory component, typically precede MI injury (Odegaard and Chawla, 2013). The smoldering immune-inflammatory response impedes infarct healing by interfering with resolution of local inflammation and delaying the reparative phase (Swirski and Nahrendorf, 2013). Although the prognosis of patients with acute MI is largely determined by the extent of myocardial tissue loss, immune-inflammation also plays a critical role in the evolution of MI-induced cardiac remodeling and may tip the balance in favor of heart failure. Kondo et al. (2013) reported that the myocardial and circulating H_2_S levels were markedly reduced in experimental models of heart failure. In addition, they found that CSE-deficient mice exhibited greater cardiac dilatation and dysfunction compared to wild-type mice after transverse aortic constriction. In contrast, cardiac-specific CSE transgenic mice maintained cardiac structure and function after transverse aortic constriction. Recently, we also demonstrated that SPRC (a novel endogenous H_2_S modulator) therapy prevented doxorubicin-induced heart failure partially via regulation of gp130/STAT3 pathways. All these data suggest that both exogenous and endogenous H_2_S exhibit cardioprotective effects in heart failure.

**FIGURE 3:**
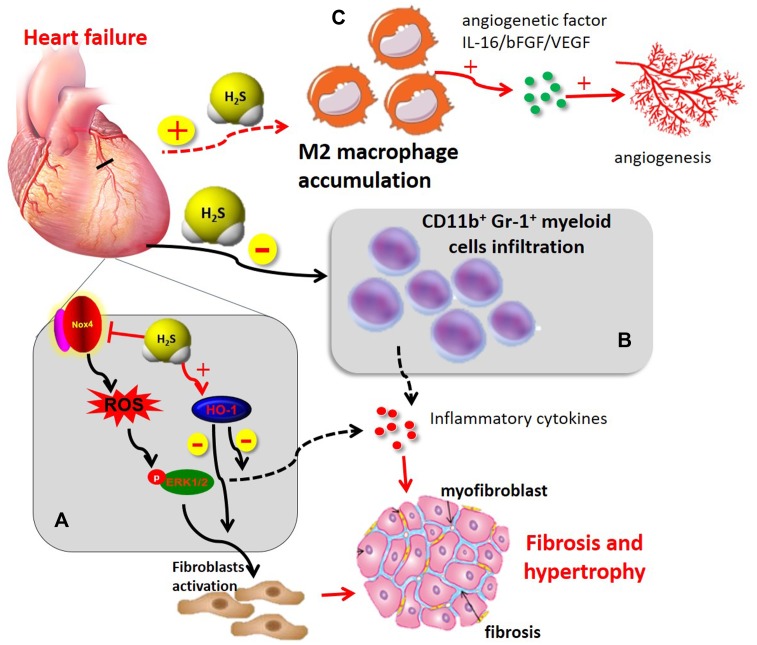
The immunomodulatory role of H_2_S in heart failure **(A)** H_2_S administration induced cardioprotection in chronic MI by improving cardiac function, attenuating myocardial fibrosis, and inhibiting chronic inflammatory mediators. This beneficial effect of H_2_S, at least in part, was associated with a decrease of Nox4/ROS/ERK1/2 signaling axis and an increase in HO-1 expression **(B)** H_2_S attenuated cardiac dilation through inhibiting recruitment of CD11b^+^Gr-1^+^ cells in infarct myocardium and inflammatory mediator release. **(C)** H_2_S induced M2 macrophage polarization and recruitment in myocardial infarction, thereby contributing to angiogenetic factor release and subsequent angiogenesis. ERK1/2, extracellular signal-regulated kinase 1/2; HO-1, heme oxygenase-1; Nox4, NADPH oxidase 4; ROS, reactive oxygen species.

In heart failure, accumulating experimental and clinical evidence points to a gradual state of immune-inflammatory activation accompanied by the progression of ventricular dysfunction with leukocyte activation and release of inflammatory mediators. For example, the inflammatory biomarker C-reactive protein, and inflammatory cytokines, such as TNF-α and IL-6, increase systemically in heart failure, and leukocytosis is associated with disease progression (Shirazi et al., 2017). Our previous study demonstrated that exogenous H_2_S administration markedly inhibited inflammatory cytokine expression in an *in vivo* model of heart failure associated with improving cardiac function and attenuating myocardial fibrosis (Pan et al., 2013). This potent beneficial pharmacological effects of H_2_S, at least partially, was associated with decreased Nox4/ROS/ERK1/2 signaling and increased HO-1 expression (Pan et al., 2013). In addition, H_2_S also inhibited chronic inflammatory responses and attenuated myocardial hypertrophy in experimental models of myocardial infarction and pressure overload induced via transverse aortic constriction (Nishida et al., 2012). Furthermore, exogenous H_2_S treatment also reduced recruitment of CD11b^+^Gr-1^+^ cells in infarct myocardium and peripheral blood and attenuated cardiac dilation in chronic ischemia-mediated infarcted myocardium in mice (Wu et al., 2017). These findings support the emerging view that H_2_S has potent immuno-inflammatory regulatory activities in ischemia-induced heart failure, resulted in reduced interstitial fibrosis, cardiac hypertrophy as well as improved overall survival (**Figure 3**).

Angiogenesis is a complex biological process that leads to increased blood flow and promotes cardiac repair and myocardium survival during heart failure. Therefore, promoting myocardial angiogenesis is a novel therapeutic strategy for the treatment of heart failure (Bao et al., 2013; Wang and Cai, 2016). In recent years, the gasotransmitter H_2_S has become apparent that it is capable of mediating angiogenesis and improving cardiac function after heart failure (Givvimani et al., 2011; Polhemus et al., 2013). In a mouse model of transverse aortic constriction-induced heart failure, chronic H_2_S treatment with diallyl trisulfide improved left ventricular remodeling and function by inducing angiogenesis *via* upregulation of VEGF and endothelial NO synthase. Additionally, the study also indicated that H_2_S upregulated the endogenous antioxidants, GPx1 and HO-1 (Polhemus et al., 2013). These results were further confirmed by our previous study that chronic H_2_S therapy with SPRC induced angiogenesis by a mechanism involving STAT-3 interacting with VEGF receptor 2 in a rat model of chronic myocardial ischemia (Kan et al., 2014). Recruitment of monocytes/macrophages to the site of injury not only promote inflammatory responses and pathological tissue remodeling but is also required for the resolution of inflammation and regenerative activities, such as angiogenesis (Lavine et al., 2014; Howangyin et al., 2016). One proposed explanation for these observations is that distinct macrophage subpopulations may mediate inflammatory (M1) and reparative (M2) macrophage activities (Lavine et al., 2014; Kolluru et al., 2015; Miao et al., 2016a). H_2_S modulates monocytes/macrophages phenotypes, which produces beneficial effects in angiogenesis in chronic ischemic diseases. Although there is no direct evidence that H_2_S modulates macrophage phenotype in heart failure, Kolluru et al. (2015) using femoral artery ligation model has demonstrated that CSE dysregulation or deficiency as well as endogenous H_2_S production have a significant effect on monocytes/macrophage recruitment and subsequent expression of angiogenetic factors (bFGF and VEGF) under ischemic conditions. Yet exogenously administering H_2_S or modulation of endogenous H_2_S production promoted monocyte/macrophage recruitment and angiogenetic factor expression, leading to angiogenesis and restored blood flow (Kolluru et al., 2015). However, the contribution of M1 vs. M2 macrophages in H_2_S-mediated angiogenetic responses in heart failure has yet to be clarified in future investigation. In addition, CD4^+^T lymphocyte deficiency delays the transition from M1 to M2 macrophages and impairs healing of the heart (Hofmann et al., 2012). Likewise, depletion of dendritic cells disturbs resolution of inflammation (Hofmann et al., 2012; Swirski and Nahrendorf, 2013). There is still lack of direct evidence for an immunoregulatory role of H_2_S on immune cell phenotypes in heart failure. Given that leukocytes play a key role in heart failure, the immunoregulatory function of H_2_S on different immune cell subsets merits further investigations.

Taken together, preclinical evidence suggests that H_2_S significantly improve cardiac function in the setting of heart failure *via* immunoregulatory activities, including modulating immune cell phenotypes, suppressing inflammatory responses and inflammatory cell infiltration, which represents a therapeutic strategy for heart failure (**Figure 3**).

## Atherosclerosis

Atherosclerosis, a vascular disease at the susceptible sites in medium and large-sized arteries, is the pathological basis of coronary heart disease and the major cause of death in developed countries. The development of atherosclerosis is a complex multifactorial process that involves vascular inflammation, VSMC proliferation and migration, thrombus formation, as well as abnormal immune responses including monocyte infiltration and differentiation, and lesion-resident macrophage conversion into foam cells (Pan et al., 2012). Through the factors that initiate plaque formation and are currently being debated, it is no longer news that atherosclerosis is more than a mere cholesterol storage disease. Immune inflammation in the pathogenesis of atherosclerosis has now gained widespread recognition (Libby and Hansson, 2015).

Recent studies have suggested that dysfunctional CSE and reduced endogenous H_2_S levels are linked to the pathogenesis of atherosclerosis (Wang et al., 2009; Mani et al., 2014). Exogenous H_2_S treatment protects rat aortic VSMC from hyperhomocysteine- or ROS-induced cytotoxicity, which is considered independent of atherogenic risk factors (Yan et al., 2006). In a genetic model of hyperhomocysteinemia, CBS^-/-^ApoE^-/-^ mice exhibited accelerated aortic atherosclerosis compared with ApoE^-/-^ mice after 6 months of age in the absence of dietary manipulation (Wang et al., 2003). Since only 2% of CBS^-/-^ApoE^-/-^ mice survived up to 6 months of age, the pathophysiological relevance of CBS^-/-^ to H_2_S metabolism in atherosclerosis is not clear (Wang et al., 2003). At the same time, CSE expression and H_2_S production were reduced during neointimal hyperplasia in carotid artery in rats, and that exogenous H_2_S treatment markedly reduced neointimal formation (Meng et al., 2007). Furthermore, Wang et al. (2009) reported that plasma H_2_S level and H_2_S production in atherosclerotic aortic tissues were decreased in ApoE^-/-^ atherosclerotic mice. Exogenous H_2_S treatment resulted in elevated plasma H_2_S level and reduced the atherosclerotic plaque size in the aortic root of ApoE^-/-^ mice, whereas DL-propargylglycine (PPG, a potent CSE inhibitor) reduced plasma H_2_S level and enlarged plaque size in the aorta (Wang et al., 2009). Furthermore, atherogenic diet feeding to ApoE^-/-^ CSE^-/-^ mice exacerbated the development of atherosclerosis compared to mice with only ApoE or CSE deficiency. Treatment of CSE^-/-^ mice with exogenous H_2_S inhibited the progression of atherosclerosis (Mani et al., 2013), which provided clear evidence that supports a protective role of H_2_S against atherosclerosis.

**FIGURE 4:**
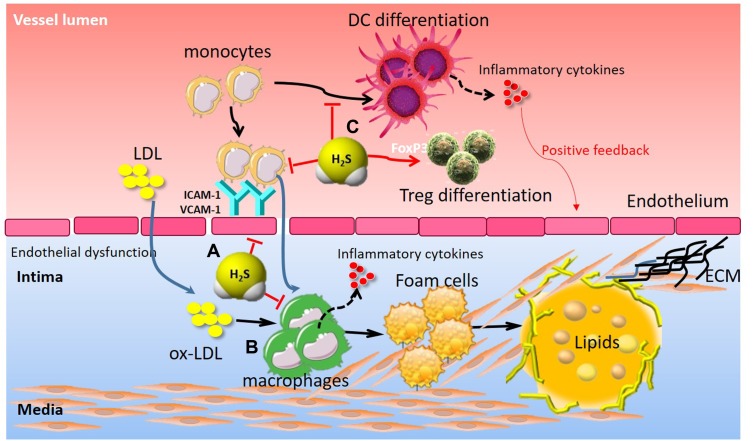
The immunomodulatory role of H_2_S in atherosclerosis. **(A)** H_2_S attenuated endothelial dysfunction, adhesion molecules (ICAM-1, VCAM-1) expression, and leukocyte adhesion. **(B)** H_2_S inhibited monocyte activation and foam cell formation, thereby contributing to inflammatory cytokine release, VSMC proliferation and subsequent atherosclerotic plaque formation. **(C)** H_2_S suppressed monocyte differentiation into inflammatory dendritic cells and induced CD4^+^Foxp3^+^ Treg cell differentiation, thereby contributing to vascular immune homeostasis. Treg, regulatory T cell; ICAM-1, intercellular adhesion molecule-1; VCAM-1, vascular cell adhesion molecule-1.

Initially, exploration of the immune-inflammatory aspects of atherogenesis focused on the intima, the site where atheromata take root. As probing has deepened, researchers have come to recognize that influence arising from all three layers of arteries can affect the pathophysiology of this disease (Libby and Hansson, 2015; Gistera and Hansson, 2017). Indeed, immune-inflammatory responses participate in atherosclerosis by modifying the arterial tree at various levels (Libby and Hansson, 2015). Accumulating evidence has indicated that H_2_S is involved in the immune-inflammatory processes in atherosclerosis in a number of preclinical models of atherosclerosis (**Figure 4**). The vascular endothelial dysfunction, characterized by the loss or dysregulation of the homeostasis, is considered an important early event in the development of atherosclerosis (Mani et al., 2014). Endothelial dysfunction is associated with increased oxidative stress, adhesion molecules expression, synthesis of inflammatory and pro-thrombotic factors, and abnormal modulation of vascular tone (Mani et al., 2014). Wang et al. (2009) demonstrated that ICAM-1 levels were significantly increased, accompanied by increased size of the atherosclerotic plaque in ApoE^-/-^ atherosclerotic mice. The ICAM-1 levels and atherosclerotic plaque were reduced in aortas of ApoE^-/-^ mice following treatment with exogenous H_2_S. The inhibitory mechanism of H_2_S on ICAM-1 expression was addressed in endothelial cells *in vitro*, where exogenous H_2_S was shown to inhibit NF-κB activation (Wang et al., 2009). We also studied the protective effects of exogenous H_2_S on TNF-α-induced dysfunction in human umbilical vein endothelial cells *in vitro* (Pan et al., 2012). Mechanically, exogenous H_2_S inhibited TNF-α-induced ICAM-1 and VCAM-1 protein expression, P-selectin and E-selectin mRNA expression, as well as monocyte adhesion to endothelial cells (Pan et al., 2012). Similarly, Feng et al. (2017) found that CSE/H_2_S pathway was significantly downregulated in the development of pulmonary vascular endothelial inflammation. H_2_S treatment could reduce pulmonary vascular pressure, relieve pulmonary vascular remodeling, inhibit pulmonary vascular endothelial cellular inflammation, and attenuate the NF-κB signaling pathway in pulmonary arterial endothelial cells. In contrast, Zanardo et al. (2006) demonstrated that suppression of endogenous H_2_S production, by CSE blockade using β-cyano-L-alanine, led to enhanced leukocyte adhesion, leukocyte infiltration, and edema formation while H_2_S donors produced opposite effects.

Macrophage is thought to play an important role in atherosclerosis by generating lipid-laden foam cells and by secreting inflammatory mediators (Moore and Tabas, 2011). Macrophage uptake of oxidized-low density lipoproteins (oxLDL) contributes to formation of lipid-laden “foam cells,” the primary component of atherosclerotic lesions (Moore and Tabas, 2011). However, H_2_S plays an inhibitory role in macrophage-derived foam cell formation. *In vitro*, Wang et al. (2013) demonstrated that oxLDL may down-regulate the CSE/H_2_S pathway, which exerts an anti-inflammatory effect on oxLDL-stimulated macrophage by suppressing JNK/NF-κB signaling. Treatment with exogenous H_2_S or upregulation of endogenous H_2_S by CSE overexpression markedly attenuated oxLDL-mediated inflammatory responses and JNK/NF-κB signaling activation (Wang et al., 2013). Du et al. (2014) further elucidated that the sulfhydration of free thiol group on cysteine 38 in NF-κB p65 served as a molecular mechanism by which H_2_S inhibited NF-κB activation in oxLDL-induced macrophage inflammation. In addition, H_2_S also abrogated oxLDL-induced macrophage foam cell formation. Mechanistically, H_2_S inhibited oxLDL-induced intracellular lipid accumulation, reduced TC, EC, and EC/TC ratio in macrophages by down-regulating expressions of CD36, scavenger receptor A and acyl-coenzyme A: cholesterol acyltransferase-1 (Zhao et al., 2011). In agreement with these findings, H_2_S treatment attenuated high glucose + oxLDL-induced foam cell formation (Xie et al., 2016). The protective effect of H_2_S can be, at least in part, attributed to Nrf2 activation via Keap1 *S*-sulfhydration at Cys151. Furthermore, H_2_S significantly inhibited macrophage accumulation and reduced the aortic atherosclerotic lesion in ApoE^-/-^ mice. However, PPG demonstrated the opposite effect: enlarging the lesion area and macrophage accumulation in the lesions of ApoE^-/-^ mice (Lin et al., 2016). H_2_S supplementation also reduced lesion zone and macrophage infiltration in diabetic LDLr^-/-^ mice. The expression of adhesion molecules and macrophages/monocyte recruitment in sub-endothelial space are critical for the initiation and development of atherosclerotic lesions. The preferential accumulation of Ly-6C^high^ monocytes in the growing atheromata relied on the C-C chemokine receptor type 2 (CCR2)-monocyte chemotactic protein-1 (CCL2), CX3CR1-CX3CL1, and CCR5-CCL5 (Gao et al., 2015), and neutralizing these axes in mice almost abolished atherosclerosis *via* reduced macrophage infiltration and increased plaque stability (Moore and Tabas, 2011). *In vivo*, Zhang et al. (2012) observed that H_2_S treatment downregulated CX3CR1 and CX3CL1 expression on macrophages through modulation of the transcription factors PPAR-γ and NF-κB. They also demonstrated that, by interfering with the CX_3_CL1/CX_3_CR1 dyad, supplementation of mice with the exogenous H_2_S reduced the development of atherosclerotic plaques (Zhang et al., 2012). They further found in a clinical study that plasma H_2_S level was markedly reduced, whereas plasma CCL2 and CX3CL1 levels were substantially increased in patients with ACS compared to patients with SAP or non-CAD patients (Gao et al., 2015). Furthermore, patients with ACS exhibited significantly higher proportions of CD14^+^CCR2^+^CX3CR1^+^ (intermediate monocytes, Mon2) and CD14^+^CCR2^-^CX3CR1^+^ monocytes (non-classical monocytes, Mon3) but a lower percentage of CD14^+^CCR2^+^CX3CR1^-^ monocytes (classical monocytes, Mon1) than patients with SAP or non-CAD did Gao et al. (2015). Lastly, they identified that plasma H_2_S level was negatively correlated with the proportion of Mon2 monocyte subsets, suggesting that impaired endogenous H_2_S synthesis in ACS may facilitate monocyte subset conversion from Mon1 to Mon2 or Mon3, leading to atherosclerotic plaque instability, and the development of ACS (Gao et al., 2015). However, the precise mechanism by which H_2_S regulates monocyte phenotypes in CAD remains to be better understood. Similarly, in methionine/choline-deficient diet-induced experimental steatohepatitis in mice, H_2_S treatment significantly prevented CX_3_CR1^+^CD11b^+^/F4^-^80^+^ cell accumulation and decreased circulating and hepatic TNF-α levels (Sutti et al., 2015). These CX_3_CR1^+^ cells were further characterized by the co-expression of inflammatory monocyte (Ly6C, CD11b) and dendritic cell (CD11c, MHCII) markers as well as by a sustained TNF-α production, suggesting that H_2_S could prevent monocyte differentiation into inflammatory monocyte-derived inflammatory dendritic cells and limit their M1 polarization (Sutti et al., 2015).

T-helper (Th) cells play a critical role in mediating adaptive immunity. During TCR activation in a particular cytokine milieu, naive CD4^+^T cells may differentiate into different lineages of Th cells, including Th1, Th2, Th17, and Treg cells. Accumulating evidence has shown that peripheral activation of Treg and subsequent recruitment to atherosclerotic plaque limit the lesion progression in experimental models by down-regulating inflammatory responses which include multiple mechanisms (Libby et al., 2013; Delgado-Maroto et al., 2017). However, minor populations of Foxp3^+^ Treg cells were found in human atherosclerotic plaques at all stages of the disease (Gistera and Hansson, 2017). Transfer of Foxp3^+^ Treg cells decreased atherosclerosis in hypercholesterolaemic mice (Gistera and Hansson, 2017). More recently, Yang et al. (2015) found that reduced H_2_S levels were responsible for impaired CD4^+^Foxp3^+^ Treg cell differentiation and function as well as immune dysfunction in mice. Treatment of H_2_S donor rescued Treg-cell-deficient phenotypes of immune dysfunction in CBS^-/-^ mice and WT Treg cell infusion could partially rescue autoimmunity in CBS^-/-^ mice (Yang et al., 2015). The immune regulatory mechanisms by H_2_S are that H_2_S affected sulfhydration of nuclear transcription factor Y subunit beta (NFYB) to control NFYB complex binding to the *Tet1* and *Tet2* promoters, forming a H_2_S-NFYB-Tet axis to regulate Treg differentiation and immune homeostasis (Yang et al., 2015). Furthermore, H_2_S can enhance TCR-dependent T cell activation and IL-2 expression. H_2_S also enhances T cell proliferation and lineage determination *via* altering cytoskeletal actin dynamics and increasing the reorientation of the microtubule-organizing center (Miller et al., 2012), suggesting that H_2_S represents a novel immunomodulatory molecule for T cell responses. Therefore, it may be a novel therapeutic approach for chronic immune-inflammatory responses in atherosclerosis *via* targeting H_2_S metabolism.

## Challenges for H_2_S Research and Future Perspectives

Since the first demonstration of the expression of H_2_S-producing enzymes in the mammalian system, there have been numerous experimental studies conducted on the role of H_2_S modulation, by ways of overexpression/inhibition of H_2_S-synthesizing enzymes or H_2_S donor, on cardiovascular homeostasis (Salloum, 2015). Mechanisms underlying H_2_S signaling have been uncovered; however, a lot of unknowns on how H_2_S influences cardiovascular homeostasis remain to be further investigated. Immune-inflammatory responses play a decisive role in different phases of cardiovascular diseases (Gistera and Hansson, 2017; Jones et al., 2017). Data from basic studies support immunoregulatory functions of H_2_S and therefore the potential of H_2_S to modulate the immune-inflammatory response to prevent cardiovascular disorders, including ischemic heart disease, atherosclerosis, heart failure, and so on. In other cardiovascular diseases such as hypertension, H_2_S has been demonstrated to play an important role (Yang et al., 2008). As arterial inflammation and immune dysregulation are involved in the pathogenesis of the disease (Smith and Ferguson, 2016) and H_2_S has been shown to maintain immune homeostasis, it could be postulated that H_2_S may play a positive role in such condition. So far literature has been limited to provide further evidence and hence is not covered in the current review, which merit future investigation and verification.

Both pro- and anti-inflammatory effects of H_2_S have been reported. In numerous studies including our studies, H_2_S has been characterized for its anti-inflammatory role (Cao and Bian, 2016; Zhou et al., 2016; Feng et al., 2017). In contrast, recent work from different groups has shown a key role of H_2_S as an inflammatory mediator (Li et al., 2011; Bhatia, 2015). These contradictory observations may result from different experimental settings and approaches, such as cell culture and/or *in vivo* disease models. However, a number of elegant studies suggest that H_2_S is a potent anti-inflammatory molecule, specifically in the cardiovascular diseases (Polhemus and Lefer, 2014; Miao et al., 2016a). Although the data are debatable, they suggest that H_2_S may be a double-edged sword and controversies are warranted to encourage future studies to better understand the biological significance of this gaseous molecule in cardiovascular homeostasis. We believe that resolving these issues would drastically advance H_2_S research.

H_2_S has a number of biological effects on cardiovascular systems. However, the molecular targets of H_2_S remains to be fully uncovered. K_ATP_ channels in many cellular systems are accountable for the effects of H_2_S. In other cases, H_2_S seems not to act on the same channels. Until now, the potential molecular targets for H_2_S are likely to include intracellular proteins or enzymes (such as p66Shc, phospholamban, protein tyrosine phosphatase 1B, mitogen-activated extracellular signal-regulated kinase 1, ATP synthase subunit α, etc.), and transcription factors (such as NF-κB, kelch-like ECH-associating protein 1, specific protein-1 and interferon regulatory factor-1, etc.) as well as membrane receptors (vascular endothelial growth factor receptor 2, insulin receptor, and epidermal growth factor receptor) in cardiovascular system (Li et al., 2011; Ge et al., 2014; Wang et al., 2016). Underlying these functions are the atomic biology, interaction between sulfur atoms and target molecules. In this regard, Tao et al. found a molecular switch in H_2_S-targeting receptor, the cysteine1024 (Cys1024)-S-S-Cys1045 disulfhide bond, in the intracellular kinase domain of vascular endothelial growth factor receptor 2 (Tao et al., 2013), which has prompted the field of H_2_S biology to a new landmark. Although remarkable progress has been made in delineating the role of the potential targets by H_2_S in cardiovascular homeostasis, one challenging question that remains in this field is the identification of more precise protein targets that mediate numerous physiological functions. These proteins propose novel targets for therapeutic intervention and drug design in cardiovascular homeostasis, which may accelerate the development and application of H_2_S related drugs in the future.

Modulation of endogenous H_2_S levels as a novel potential therapeutic strategy for cardioprotection in patients undergoing cardiovascular disorders and the changed H_2_S-producing enzymes expression/activities have been directly related to the endogenous H_2_S generation. Meanwhile, the three H_2_S-generating enzymes have been broadly localized in cardiovascular system (Pan et al., 2012). Downregulation of the three H_2_S-producing enzymes is associated with chronic cardiovascular pathologies (Kanagy et al., 2017; Merz et al., 2017). Unfortunately, the H_2_S-producing enzymes responsible for H_2_S production and biological function in cardiovascular homeostasis are not clear and consistent in current literature (Kuo et al., 2016; Li et al., 2016). Meanwhile, the roles of H_2_S degradative enzymes ETHE1, SQR and CDO in cardiovascular immune homeostasis remain largely unexplored and merit further investigation (Rose et al., 2017).

Over the last decade, considerable evidence has been collected, which points to a functional role for H_2_S in cardiovascular homeostasis, representing a novel promising therapeutic strategy for cardiovascular diseases. However, the majority of the cardiovascular studies involving H_2_S have been investigated and established in healthy, juvenile, and small animals, making them far removed from the clinical setting of the typical cardiovascular diseases. Meanwhile, improved understanding on the protective actions of H_2_S, together with rapid development of novel H_2_S donors (Polhemus and Lefer, 2014; Hackfort and Mishra, 2016; Zheng et al., 2017), has raised heightened enthusiasm for the translational studies. Currently, there are three cardiovascular H_2_S trials on clinicaltrials.gov. Therefore, it will be expedient to move one step forward to confirm rigorously the therapeutic effects of H_2_S in larger animal models before making a complete transition to the clinic.

As with the development of small molecule H_2_S donors, there are organ-specific issues that need to be considered. Given its ubiquitous nature, it is not surprising that H_2_S has important functions in a wide range of physiological and pathophysiological processes. H_2_S delivery will produce a wide range of biological activities, including unwanted side effects. Therefore, the speed and amount of H_2_S release from different donors should be controllable. Otherwise, a novel H_2_S donor that could specifically target an organ system would alleviate undesirable effects. Because the cardiovascular system is a circulatory system, the cardiovascular-specific delivery of H_2_S maintained cardiovascular homeostasis is one of the key challenges being explored in the field.

## Conclusion

H_2_S is a ubiquitous gasotransmitter and plays a critical role in immune homeostasis in cardiovascular disorders. Significant changes of endogenous H_2_S levels (change of H_2_S-producing enzyme expression or its activity) have been clearly correlated to immune-inflammatory responses in a variety of cardiovascular diseases. We have summarized the latest knowledge on the immune-inflammatory modulatory functions of H_2_S in cardiovascular diseases and discussed the possible cellular and molecular mechanisms by which it exerts cardiovascular protective actions as well as its therapeutic potential for cardiovascular diseases. Although the molecular targets of H_2_S remain to be fully elucidated, considerable evidence has demonstrated that H_2_S is a novel immune-modulator in cardiovascular homeostasis. Insights into the molecular targets of H_2_S in immune-inflammatory processes may help better understanding of the pathophysiology of these diseases.

## Author Contributions

X-HL and Y-ZZ designed the subject content of the review article. L-LP, MQ, and X-HL conducted initial search of literature, drafted the manuscript, and prepared the figures and tables. X-HL and Y-ZZ had primary responsibility for final content. All authors read and approved the final manuscript.

## Conflict of Interest Statement

The authors declare that the research was conducted in the absence of any commercial or financial relationships that could be construed as a potential conflict of interest.

## Abbreviations

3-MP: 3-mercaptopyruvate
3-MST: 3-mercaptopyruvate sulfurtransferase
AAR: area at risk
ACS: acute coronary syndrome
CAD: coronary artery disease
CAT: cysteine aminotransferase
CBS: cystathionine-β-synthase
CDO: cysteine dioxygenase
CO: carbon monoxide
CSE: cystathionine-γ-lyase
DAO: D-amino acid oxidase
EC: esterified cholesterol
ETHE1: ethylmalonic encephalopathy protein 1
ERK1/2: extracellular signal-regulated kinase 1/2
Foxp3: forkhead box protein P3
H_2_S: hydrogen sulfide
HO-1: heme oxygenase-1
ICAM-1: intercellular adhesion molecule-1
iNOS: inducible nitric oxide synthase
IL: interleukin
MI: myocardial infarction
MI/R: myocardial ischemia/reperfusion
NF-κB: nuclear factor-κB
NLRP3: nucleotide-binding domain, leucine-rich-containing family, pyrin domain-containing-3
NO: nitric oxide
Nox4: NADPH oxidase 4
oxLDL: oxidized LDL
PPG: DL-propargylglycine
PPAR-γ: proliferators-activated receptor-γ
ROS: reactive oxygen species
SAP: stable angina pectoris
SQR: sulfur:quinone oxidoreductase
SPRC: *S*-propargyl-cysteine
STAT3: signal transducer and activator of transcription 3
TC: total cholesterol
TCR: T-cell receptor
TNF-α: tumor necrosis factor-α
Tregs: regulatory T cells
VCAM-1: vascular cell adhesion molecule-1
VEGF: vascular endothelial growth factor
VSMC: vascular smooth muscle cell
